# Short-term economic evaluation of physical activity-based corporate health programs: a systematic review

**DOI:** 10.1093/joccuh/uiae002

**Published:** 2024-01-05

**Authors:** Lorenzo Bonatesta, Stefano Palermi, Felice Sirico, Mario Mancinelli, Pierpaolo Torelli, Ettore Russo, Giada Annarumma, Marco Vecchiato, Frederik Fernando, Giampietro Gregori, Josef Niebauer, Alessandro Biffi

**Affiliations:** Med-Ex, Medicine & Exercise, Medical Partner Scuderia Ferrari, 00187 Rome, Italy; Med-Ex, Medicine & Exercise, Medical Partner Scuderia Ferrari, 00187 Rome, Italy; Public Health Department, University of Naples Federico II, 80131 Naples, Italy; Med-Ex, Medicine & Exercise, Medical Partner Scuderia Ferrari, 00187 Rome, Italy; Public Health Department, University of Naples Federico II, 80131 Naples, Italy; Med-Ex, Medicine & Exercise, Medical Partner Scuderia Ferrari, 00187 Rome, Italy; Med-Ex, Medicine & Exercise, Medical Partner Scuderia Ferrari, 00187 Rome, Italy; Med-Ex, Medicine & Exercise, Medical Partner Scuderia Ferrari, 00187 Rome, Italy; Med-Ex, Medicine & Exercise, Medical Partner Scuderia Ferrari, 00187 Rome, Italy; Sports and Exercise Medicine Division, Department of Medicine, University of Padova, 35128 Padova, Italy; Med-Ex, Medicine & Exercise, Medical Partner Scuderia Ferrari, 00187 Rome, Italy; Med-Ex, Medicine & Exercise, Medical Partner Scuderia Ferrari, 00187 Rome, Italy; University Institute of Sports Medicine, Prevention and Rehabilitation, Paracelsus Medical University, 5020 Salzburg, Austria; Med-Ex, Medicine & Exercise, Medical Partner Scuderia Ferrari, 00187 Rome, Italy

**Keywords:** corporate health program, physical activity, ROI, economic evaluation, employee

## Abstract

**Objectives:** Corporate health programs (CHPs) aim to improve employees’ health through health promotion strategies at the workplace. Physical activity (PA) plays a crucial role in primary prevention, leading many companies to implement PA-based CHPs. However, there is limited examination in the scientific literature on whether PA-based CHPs (PA-CHPs) lead to economic benefits. This systematic review aimed to summarize the available literature on the economic aspects of PA-CHPs.

**Methods:** A systematic review was conducted to identify studies focused on PA-CHPs targeting healthy sedentary workers and reporting at least one economic outcome, such as return on investment (ROI), costs, or sick leave.

**Results:** Of 1036 studies identified by our search strategy, 11 studies involving 60 020 participants met the inclusion criteria. The mean (±SD) cost per capita for PA-CHPs was estimated as 359€ (±238€) (95% CI, 357-361€). In 75% of the studies, the net savings generated by PA-CHPs in 12 months were reported, with an average of 1095€ (±865€) (95% CI, 496-1690€). ROI was assessed in 50% of the included studies, with an average of 3.6 (±1.41) (95% CI, 2.19-5.01).

**Conclusions:** In addition to promoting a healthy lifestyle, PA-CHPs have the potential to generate significant economic returns. However, the heterogeneity among the existing studies highlights the need for standardization and accurate reporting of costs in future research.

## Teaser

**Key points**
 

- Even if many companies adopt these programs, the scientific literature does not examine whether physical activity-based corporate health programs (PA-CHPs) lead to economic benefits.
- Our systematic review shows that PA-CHPs, as stand-alone or combined with lifestyle changes, can generate a net saving of 1095€ (on average) and a return on investment of 3.6.
- In addition to promoting a healthy lifestyle, PA-CHPs have the potential to generate a significant economic return.

## 1. Introduction

In recent years, primary prevention programs targeting chronic noncommunicable diseases (NCDs) have gained significant attention. These programs aim to mitigate the negative impact of NCDs on the general asymptomatic population.^1^ Within this context, corporate health programs (CHPs) sponsored by employers have emerged as a means to improve employee health and enhance productivity while reducing costs associated with sickness absence.^2^^,^^3^

CHPs primarily focus on addressing modifiable risk factors such as tobacco use, high fat intake, and physical inactivity, which are known to contribute to sick leave of workers.^4^^,^^5^ Among these, physical activity (PA) has been extensively studied and proven to be effective in reducing NCD risk factors, preventing diseases, and generating cost savings for individuals and health care systems.^6^^–^^12^

Sedentary workers spend 70% of their working time in desk-based activites: this behavior has been linked to type 2 diabetes, cardiovascular diseases, cancer, and premature death.^13^ Moreover, the impact of physical inactivity-related preventable illnesses has been estimated as 4.6% of the total economic expenditure of a Western country’s health care system.^14^

However, the scientific literature currently lacks standardized guidelines for CHPs, resulting in ongoing debates regarding their preventive efficacy.^15^^–^^18^ Although the inclusion of PA in CHPs shows promise,^3^^,^^19^^–^^21^ the lack of systematic methodological analysis weakens the scientific significance of these findings. Moreover, despite the growing implementation of these programs, there remains a significant gap in the literature regarding their economic implications.

Therefore, this study aimed to systematically analyze the effects of PA-focused CHPs (PA-CHPs) among sedentary workers on specific outcomes, including return on investment (ROI), cost savings, sick leave days, and overall costs. By addressing these specific outcomes, this study aimed to contribute to the understanding of the impact of PA-CHPs in the workplace.

**Figure 1:**
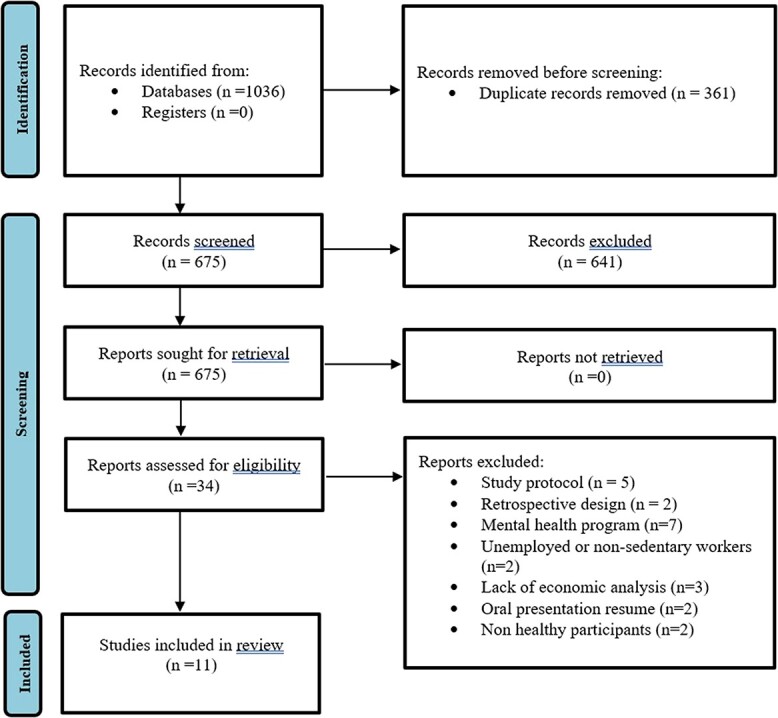
>Preferred Reporting Items for Systematic reviews and Meta-analyses (PRISMA) flowchart.

**Figure 2:**
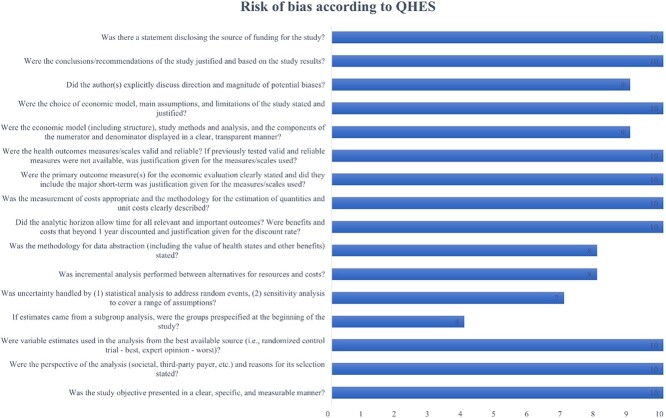
>Risk-of-bias graph.

**Figure 3:**
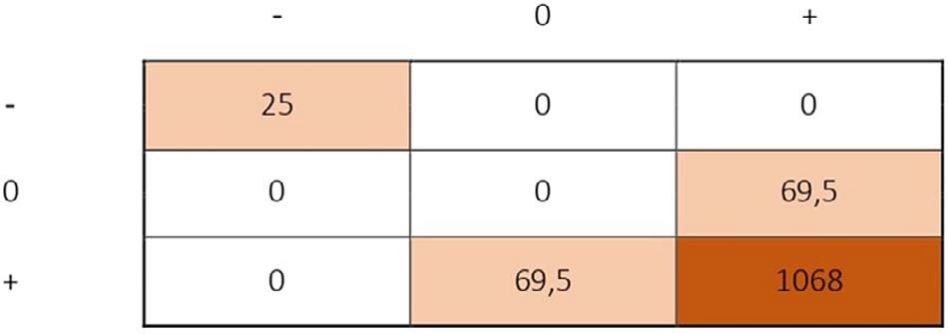
>Hierarchical matrix.

## 2. Methods

### 2.1. Data sources

A systematic review of the available literature was conducted following the Consolidated Health Economic Evaluation Reporting Standards (CHEERS) 2022 version^22^ and the Preferred Reporting Items for Systematic reviews and Meta-analyses (PRISMA).^23^ A complete protocol was prospectively registered in the Centre for Reviews and Dissemination’s Prospective Register of Systematic Reviews (PROSPERO), with the protocol number CRD42022343412.

### 2.2. Search strategy

A comprehensive search strategy was developed using previously published reviews on the topic as a source for relevant terms.^3^^,^^19^^,^^20^ The search was conducted in the following databases: PubMed, Cochrane Central, LILACS, IBECS, Web of Science, Cumulative Index to Nursing and Allied Health Literature (CINAHL), PsycINFO, and Scopus. The search period extended from inception until November 2022. In addition, manual searches of published and unpublished studies (including conference abstracts, textbooks, and “gray” literature) were performed, and reference lists of retrieved articles were screened. The search strategy used the following keywords: ((“Costs and Cost Analysis”[Mesh] AND “Health Promotion”[Mesh] AND “Workplace”[Mesh]) AND (“Clinical Trial” [Publication Type])) OR ((“cost effectiveness” OR “cost analysis” OR “economic” OR “return on investment”) AND (“work intervention$” OR “workplace intervention$” OR “occupational intervention” OR “corporate wellness” OR “work-site” OR “work setting” OR “work environment”) AND (trial OR random*)).

### 2.3. Study selection

To ensure the highest level of evidence, only randomized controlled trials (RCTs) and nonrandomized controlled trials (NRCTs) were included. No language restrictions were applied.

The Patient Intervention Comparison Outcome and Study design (PICOS) framework was used to guide the study question^24^:

P—population: healthy sedentary workers, defined as office desk-based participants that predominantly work in static positions and are without any known diseases.

I—intervention: PA-CHP that included at least 1 component of PA such as steps per day goal, active breaks, or supervised exercise sessions.

C—comparison: control group of workers not involved in PA-CHP (defined as “Sit & Wait”).

O—outcome: analyze at least 1 of the following: ROI, costs, or sickness absence.

The selection process was conducted in 2 stages. Citations from the search strategy were imported into Rayyan^25^ for screening by 2 investigators (L.B. and S.P.) independently reviewing and eliminating duplicates. Any disagreements were resolved by a third investigator (P.T.). The references of relevant reviews and gray literature were also screened, and the inclusion decisions were made by B.L. A fourth investigator (S.P.) assessed their possible inclusion. To enhance agreement between investigators, a pilot screening was conducted on the first 100 screened trials, and the selection criteria were adjusted accordingly (by Cohen’s *k* index if lower than 0.61, defined as substantial agreement^26^). Consistency among investigators’ judgments was calculated for the entire selection process.

### 2.4. Data extraction

Two blinded investigators (L.B. and P.T.) independently screened the results, and their assessments were cross-checked point by point by another investigator (S.P.). A machine learning tool called Robot Review^27^ was used for third-level checking. Data were extracted following the PICOS method^24^ and the Template for Intervention Description and Replication (TIDieR)^28^ framework. The extracted data included subject characteristics (occupation, educational level, age, sex, body mass index), intervention characteristics (type of PA-CHP, duration, adherence strategy), outcome measures (ROI, costs, sick leave, and incremental cost-effectiveness ratio other economic evaluations), and study design information (follow-up, randomization, drop-out rate). The CHEERS checklist^22^ was used as a framework.

### 2.5. Quality assessment

The quality of evidence was evaluated through the Grading Recommendation Assessment, Development, and Evaluation (GRADE)^29^ approach, which considers various factors such as risk of bias, inconsistency, indirectness, imprecision, publication bias, magnitude of effect, dose–response gradient, and plausible residual confounders. We realized the assessment through the Cochrane platform Grade pro GDT.^30^ The risk of bias was assessed using the Quality of Health Economic Analysis (QHES)^31^ tool, with two investigators (L.B. and S.P.) independently rating the studies and any disagreements resolved through discussion. The QHES tool^32^ provides a numeric score for each trial, with a score of >75 indicating high quality.

### 2.6. Data analysis

The extracted data were analyzed using STATA software ( v.12, StataCorp, College Station, TX, USA). Mean, SD, and 95% CIs were calculated for each study, based on the available data types. ROI was calculated using the formula (Benefit − Cost)/Cost,^33^ for studies where this value was not provided. A hierarchical matrix was created following CHEERS guidelines,^34^ and a weighted hierarchical matrix was developed based on published guidelines,^35^ with the weighting of the effects calculated using the formula $\Big(\frac{n}{N}\times net\ benefit$),^35^ where *n* represents the sample of the individual study and *N* is the total sample size of included studies. Studies with different time points were combined into a single effect size for the analysis.^36^ All values were converted to euros for the year 2023, and discount rates were applied using online calculators provided by government websites according to the Organization for Economic Cooperation and Development.^37^^–^^41^

### 2.7. Reporting of economic outcome

All economic outcomes in this review were expressed in costs or savings per capita. Studies that reported savings or costs for the entire sample were divided by the number of participants in the intervention group.

## 3. Results

### 3.1. Study selection

Initially, 1036 articles were identified, and after removing duplicates the titles and abstracts of 675 studies were screened. Following the application of inclusion and exclusion criteria, 34 studies were retrieved in full text. Of these, 24 studies were excluded, resulting in the inclusion of 11 studies. A flowchart illustrating the selection process is presented in Figure 1, following the PRISMA guidelines.^42^

### 3.2. Risk-of-bias assessment

The agreement between 2 investigators (L.B. and S.P.) during the risk-of-bias assessment process was 0.90 (according to the *k* index), indicating “almost perfect agreement” according to the Cochrane Handbook for Systematic Reviews.^43^

### 3.3. Quality of the included studies

The quality of included trials ranged from 57 points^44^ to 99 points,^44^ and the mean (±SD) value of included studies was 80.25 (±9.03). Based on the QHES guideline score interpretation^32^ (Figure 2), 80% of included studies^44^^–^^52^ were considered “high quality,” whereas the remaining 20% were classified as “fair quality.”^53^^,^^54^

### 3.4. Characteristics of included studies

Among the included studies, 92% were RCTs,^44^^–^^47^^,^^49^^–^^52^^,^^54^ and 2 studies employed cluster randomization,^46^^,^^53^ whereas 1 study^48^ was an NRCT. The majority of the studies were conducted in the Netherlands,^46^^,^^47^^,^^49^^,^^52^^,^^53^ 20% were located in the United States,^45^^,^^54^ and the remaining studies were conducted in Australia,^44^ England,^50^ and Northern Ireland.^48^ The time frame of the studies varied, with most studies taking place between 2010 and 2020.^44^^,^^48^^,^^50^^,^^52^^,^^54^ Half of the studies employed a 12-month follow-up period,^44^^,^^45^^,^^47^^,^^52^ whereas the remainder analyzed a 24-month follow-up.^49^^,^^51^^,^^53^^,^^54^

### 3.5. Characteristics of the sample

The included trials enrolled a total of 60 020 participants, with a mean (±SD) age of 44.2 (±2) years.^44^^–^^53^ The mean (SD) age of the study sample was not substantially different from the control group in all included studies, that is, 44.2 (±3.5) years. Information on body mass index (BMI) was reported in less than half of the included studies, with all participants classified as overweight if their BMI was ≥25 kg/m^2^.^45^^,^^46^^,^^48^^,^^49^

### 3.6. Characteristics of the interventions

In 40% of the included studies, PA promotion was employed as a standalone strategy,^44^^,^^47^^,^^48^^,^^54^ whereas in the remainder it was combined with other health promotion components such as dietary interventions,^45^^,^^49^^,^^51^^,^^53^ ergonomics,^46^^,^^52^ psychological support, or cardiovascular health promotion.^45^ Face-to-face sessions were the most common delivery mode (50% of studies),^46^^,^^47^^,^^49^^,^^51^^,^^52^ followed by online-based interventions,^45^^,^^53^ whereas the remainder did not provide such information. Additional materials to remind workers to stay active, such as pedometers or email-based reminders, were provided in half of the included studies,^44^^–^^46^^,^^48^^,^^50^^,^^53^ and 20% of the studies offered standing desks and bike working stations.^46^^,^^50^ The providers of programs were specified in just two studies (a physiotherapist and a physician, respectively^46^^,^^54^), whereas in one trial^54^ a wellness vendor was involved. All included studies had a control group defined as “Sit & Wait,” in which no intervention was delivered.

### 3.7. Characteristics of economic evaluations

The economic analyses considered different perspectives, with 42% of the studies using the societal perspective,^44^^,^^49^^,^^51^^,^^53^^,^^54^ 27% considering the employer’s perspective,^45^^,^^47^^,^^48^ and the remaining studies presenting analyses from both perspectives.^46^^,^^52^ The source of economic data was declared in 36% of the studies,^46^^,^^47^^,^^52^^,^^54^ with most of the data extraction obtained from company records. Incremental analysis was conducted in 33% of the studies,^44^^,^^46^^,^^49^^,^^52^ whereas the sensitivity analysis was performed in half of them.^44^^,^^46^^,^^47^^,^^49^^,^^51^^,^^52^ Only three studies reported the economic model used, including the friction cost method,^53^ bootstrapped method,^52^ and Markov method.^44^ The transparency of the formulas used to estimate the benefits of the interventions was provided in 54% of the studies.^46^^,^^47^^,^^49^^,^^51^^–^^53^ The funding source was disclosed in 64% of the included studies,^44^^,^^45^^,^^47^^–^^49^^,^^52^^,^^53^ with 2 studies being financed by private foundations,^47^^,^^52^ and the remaining studies by national sources of funding.

**Table 1:** Characteristics of the included studies.

**Study**	**Country**	**Rate of dropout**	**Physical-activity based intervention**	**Additional material**
Allen et al, 2012	USA	C: 1%; I: 1%	Lifestyle education focused on cardiovascular, nutritional, and physical activity. Healthy foods were also provided	Pedometers (Digi-Walker SW-401, Lees Summit, MO, USA) and instructions to wear them during waking hours; the objective was 10 000 daily steps
Ben et al, 2020	The Netherlands	C: 0.9%; I: 24%	PT explained the risk of sedentary behaviors and discussed solutions; during the second session PT answered questions and discussed barriers to an active lifestyle	Sit-stand desks, cycling workstations, and office sit balls, and an activity/sitting self-monitoring tracker with self-help program booklet
Bernaards et al, 2010	The Netherlands	C: 0.8%; I: 0.8%	ND	No
Dallat et al, 2013	Northern Ireland	C: 24%; I: 24%	Physical activity card was used to incentivize participants to stay active	A card in which the physical activity level and time of activity were recorded; participants could check their levels through an application (using the activPAL3)
Gao et al, 2018	Australia	C: 0%; I: 27%	Physical activity card was used to incentivize participants to stay active	A card in which the physical activity level and time of activity were recorded, participants could check their levels through an application (using the activPAL3)
Gussenhoven et al,2012	The Netherlands	C: 28%; I: 27%	Counseling about how to keep a healthy and active lifestyle; barriers and possible solutions were discussed with the counselor	All groups, including the control group, received self-help materials about physical activity and nutrition published by The Netherlands Heart Foundation
Munir et al, 2020	England	C: 33%; I: 17%	Participants received supporting behavior change strategies including education, behavioral feedback, self-monitoring and prompt tools, quarterly coaching sessions, and information	Height-adjustable workstation; motivational posters
Proper et al, 2004	The Netherlands	C: 0.5%; I: 28%	The counseling was mainly aimed at the promotion of physical activity and healthy dietary habits using standardized protocols and the individual’s stage of behavior change	No
Robroek et al, 2012	The Netherlands	C: 38%; I: 42%	Extensive computer-tailored advice on participants’ self-reported physical activity and fruit and vegetable intake. The electronically generated advice included personal and action feedback, considering perceived barriers for participants not meeting the guidelines	Monthly email messages during the first 12 months of the study that focused on physical activity and nutrition
Song et al, 2021	USA	ND	Multicomponent workplace wellness program was focused on key topics in prevention and wellness, including nutrition, physical activity, and stress reduction	No
Van Doghen et al, 2017	The Netherlands	C: 0.9%; I: 20%	Motivational interview aimed to increase physical activity and monitoring; environmental modification in the coffee break area	No

C, control group; I, intervention group; ND: not declared; PT, physiotherapist.

**Figure 4:**
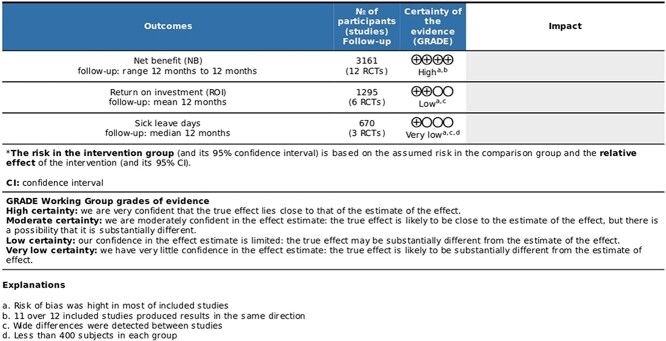
>Summary of findings by GRADE system.

### 3.8. Characteristics of economic outcomes

The most frequently reported economic outcome was the direct cost of the interventions,^44^^–^^53^ with the mean (±SD) cost for PA-CHP being 359€ (±238€) (95% CI, 201 to 507€). The net benefit of the PA-CHP was described in 64% of the studies,^46^^,^^47^^,^^49^^–^^54^ with the majority reporting a positive benefit, although the values varied across studies, ranging from 130€^49^ to 2079€.^53^ The mean (±SD) benefit produced by the intervention was 1095€ (±865€) (95% CI, 496 to 1690€). Only 1 study^47^ reported an expense of 303€ in the intervention group. ROI was provided in 43% of the included studies,^46^^,^^50^^–^^53^ with all reported values being positive but varying in magnitude, ranging from 0.46^50^ to 16.5^52^; in the rest of studies the ROI was 2.56,^50^ 3.24,^47^ and 4.99.^53^ According to the CHEERS guidelines,^34^ a hierarchical matrix was constructed based on all comparisons presented in the included studies (Figure 3), highlighting the strong preference for the intervention compared with the control group. Investigators declared to have included every cost assumed to accrue from the health promotion programs, and these data were variable depending on which intervention was included. Relevant materials or services provided by companies are stated in Table 1.

### 3.9. Effects on sick leave

Two studies assessed sick leave, reporting the number of days taken off work.^47^^,^^51^ In both studies, the PA-CHP resulted in a reduction in sick leave days compared with the control group, with reductions of 4 and 7 days, respectively.

### 3.10. Quality of the evidence

Based on the GRADE approach (Figure 4), there is high-quality evidence supporting the finding that PA-CHP produces a positive net benefit. The evidence quality for positive ROI was rated as low, and the evidence for the reduction in sick leave was rated as very low.

## 4. Discussion

This study aimed to systematically review the health-related economic aspects of RCTs and NRCTs comparing comprehensive workplace programs with at least one component of physical activity with a control group that did not receive any intervention (“Sit & Wait” control group). Our findings indicate that PA-CHPs can generate significant economic returns.

Notably, these results are based on analyses of studies conducted in different countries (Europe, Australia, United States), suggesting a global interest in this topic. The included studies encompassed over 60 000 participants with an average age of approximately 40 years, a majority of whom were overweight. This highlights the concerning prevalence of excess weight among sedentary workers.^55^

According to the findings of this review, programs that include both PA promotion and healthy dietary habits promotion are more likely to provide positive economic outcomes. The implementation of such programs was found to be more effective if the content was conveyed during counseling sessions as opposed to booklets or informative emails Of note, there was no correlation between the amount of money allocated to PA-CHP and ROI or net saving, which highlights the necessity to plan an effective and precise health promotion strategy. As suggested by some authors,^15^ key components may include a multidisciplinary care model, a clinical program, bidirectional referrals and communication with the healthcare system, the use of integrative techniques, and continuous quality improvement. However, it is important to consider the care of different vulnerable occupational groups, the high level of complexity of these initiatives, and the interdisciplinary work required for successful implementation. The choice of PA to be included in a CHP is also a major point of discussion. In the included studies, PA programs primarily focused on increasing daily steps^44^^,^^45^^,^^48^ or overall PA levels of employees.^46^^,^^47^^,^^49^^–^^54^ However, none of the studies implemented a medically supervised PA program. Some studies also incorporated nutritional management,^45^^,^^49^^,^^53^^,^^54^ which can be beneficial as there is a positive relationship between PA and diet in controlling risk factors.^56^ A notable limitation of the included studies is the lack of information about the manager of the CHP, which represents a significant gap in the literature.

The quality of evidence for the net benefit produced by PA-CHPs was high, but it was low for positive ROI and very low for reduction in sick leave days compared with the control group without any intervention. The wide variation across different studies influenced the quality of evidence for ROI and sick leave days, whereas the small sample sizes (<400 participants) in some studies contributed to the downgrading of evidence according to GRADE guidelines.^29^ No relationship between the risk of bias and the study results was found, suggesting that the risk of bias may not significantly affect the results of our analysis.

Although, to our knowledge, this study is the first attempt to systematically collect health economic data on PA-CHPs, other nonsystematic reviews and systematic economic reviews have been conducted on this topic. However, a key distinction of our review is the inclusion of a wide range of interventions in the same analysis. For instance, the study by Vargas-Martínez et al^57^ may be considered the first attempt to systematically report health economic outcomes related to CHPs, including not only PA-CHPs but also lifestyle and nutritional counseling. The authors concluded that CHPs were effective in generating savings. A direct comparison between our findings and their study was not possible due to methodological differences, because they included a wide range of dietary, PA, ergonomic, or psychosocial interventions and results were pooled into one analysis. Another scoping review by Unsal et al^3^ included various types of CHP and reported an ROI of 7, which aligns with the ROI findings of our study, ranging between 2.56 ^51^ and 4.99.^53^ However, when absenteeism, presenteeism, and health care utilization were included in the calculation, the ROI dropped to 4.^3^ This could partially explain the heterogeneity between studies, because of the calculation method used by the authors. Indeed, for all included studies, an ROI recalculation according to the formula, (benefit − cost)/cost, was realized^33^ to ensure that all declared costs were considered. This highlights the crucial role of cost considerations in determining the ROI.

On the other hand, PA-CHPs demonstrated a net benefit in favor of the intervention group, suggesting that they could be a viable investment for companies. This aspect is of great importance from a company’s perspective, as cost savings are one of their main concerns. To make CHPs suitable and sustainable from the employers’ perspective, several factors should be considered. For instance, the dropout rate can serve as an indicator of acceptability, which aligns with the acceptability of clinical PA programs.^58^ Additionally, it is important to include low-risk workers in CHPs, as they are often excluded from health promotion programs based on their satisfactory fitness profiles, which are perceived as not requiring further encouragement or improvement. However, this represents a limitation in implementing such projects within companies.^15^

Despite our efforts to develop a rigorous methodology, this systematic review is not without limitations. Two authors did not respond to our requests for more detailed information about their calculations. Due to substantial methodological differences among the included studies, a meta-analysis was not feasible according to the Cochrane handbook.^43^ Regarding the limitations of the included studies, the generalizability of the results may be affected by selection bias, as participants voluntarily chose to participate in the PA program. Although our review focuses on the short-term economic evaluation of PA-CHPs, we recognize the potential long-term health benefits of these programs, such as the prevention of chronic diseases. However, the limited follow-up duration of the included studies constrains our ability to assess these long-term outcomes. We advocate for future research to include extended follow-up periods, enabling a more comprehensive evaluation of the impact of PA-CHPs on chronic disease prevention and overall long-term health benefits. Allthough we acknowledge the importance of detailed demographic information in understanding the impact of PA-CHPs, the available data from the included studies were limited primarily to age and BMI. Future research in this area would benefit from a more thorough demographic analysis, allowing for a nuanced understanding of how factors like socioeconomic status, gender, and other demographic variables influence the economic outcomes of PA-CHPs. Another limitation is related to the calculation of health economic outcomes, as the count of benefits is closely tied to the costs considered, and different studies may yield significantly different benefits depending on the costs included in their calculations.

Future trials in this field should focus on systematically describing the costs included in ROI calculations to enhance comparability and provide a clearer understanding of the economic outcomes. Moreover, efforts should be made to address the identified gaps in the literature and further explore the optimal components and management of CHPs, including their suitability and sustainability from the employers’ perspective.

### 4.1. Conclusions

Overall, this review demonstrates the potential of PA-CHPs, as stand-alone interventions or combined with lifestyle behavior change, to generate economic benefits while addressing the health needs of sedentary workers. Implementing effective CHPs has the potential to improve employees’ well-being, reduce health care costs, and contribute to the overall productivity and success of companies.

To apply a successful PA-CHP it is not necessary to use expensive tools, even if a major investment in PA-CHP would provide better economic benefit. The popularity of these programs will grow in the future, in line with other types of PA programs.

## Funding

All authors declare no source of funding relevant to this study.

## Author contributions

L.B., S.P., and F.S. designed the study, contributed to the data acquisition strategy, and performed the preliminary analysis. M.M., P.T., F.F., and E.R. conducted the literature search, performed the initial selection of articles, and contributed to the data extraction. G.A. coordinated the study and performed the quality assessment of the included studies. M.V. contributed to the data analysis and interpreted the data. F.F. drafted the initial manuscript. G.G. critically revised the manuscript for important intellectual content. J.N. provided methodological advice and helped with the interpretation of the data. A.B. acted as the study guarantor, contributed to the design of the study, revised the manuscript for important intellectual content, and approved the final version of the manuscript. All authors have read and agreed to the published version of the manuscript.

## Conflicts of interest

None declared.

## Data availability

The data underlying this article will be shared on reasonable request to the corresponding author.
